# Identifying the DNA methylation preference of transcription factors using ProtBERT and SVM

**DOI:** 10.1371/journal.pcbi.1012513

**Published:** 2025-05-13

**Authors:** Yanchao Li, Quan Zou, Qi Dai, Antony Stalin, Ximei Luo

**Affiliations:** 1 School of Information and Software Engineering, University of Electronic Science and Technology of China, Chengdu, Sichuan, China; 2 Institute of Fundamental and Frontier Sciences, University of Electronic Science and Technology of China, Chengdu, Sichuan, China; 3 Yangtze Delta Region Institute (Quzhou), University of Electronic Science and Technology of China, Quzhou, Zhejiang, China; 4 College of Life Science and medicine, Zhejiang Sci-Tech University, Hangzhou, Zhejiang, China; Penn State University: The Pennsylvania State University, UNITED STATES OF AMERICA

## Abstract

Transcription factors (TFs) can affect gene expression by binding to certain specific DNA sequences. This binding process of TFs may be modulated by DNA methylation. A subset of TFs that serve as methylation readers preferentially binds to certain methylated DNA and is defined as TFPM. The identification of TFPMs enhances our understanding of DNA methylation’s role in gene regulation. However, their experimental identification is resource-demanding. In this study, we propose a novel two-step computational approach to classify TFs and TFPMs. First, we employed a fine-tuned ProtBERT model to differentiate between the classes of TFs and non-TFs. Second, we combined the Reduced Amino Acid Category (RAAC) with K-mer and SVM to predict the potential of TFs to bind to methylated DNA. Comparative experiments demonstrate that our proposed methods outperform all existing approaches and emphasize the efficiency of our computational framework in classifying TFs and TFPMs. Cross-species validation on an independent mouse dataset further demonstrates the generalizability of our proposed framework In addition, we conducted predictions on all human transcription factors and found that most of the top 20 proteins belong to the Krueppel C2H2-type Zinc-finger family. So far, some studies have demonstrated a partial correlation between this family and DNA methylation and confirmed the preference of some of its members, thereby showing the robustness of our approach.

## Author summary

DNA methylation affects gene expression by regulating the transcription process. Recent studies have shown that some transcription factors have specific preferences for binding to methylated DNA. Identifying these preference is fundamental to studying the regulatory processes of DNA methylation. Traditional experimental identification methods are time-consuming and labor-intensive, highlighting the need for computational approaches. However, previous methods have two problems: efficiency and robustness. We have developed a new two-step method for this. First, the transcription factors are identified through the fine-tuned ProtBERT model, then the information redundancy is reduced through the Reduced Amino Acid Category method, the protein sequences are expressed in a k-mer manner, and finally the SVM model is used to classify them. Comprehensive evaluation shows that our method significantly outperforms previous computational methods in most metric. We further validated the approach on an independent mouse dataset via two complementary experiments, consistently demonstrating superior results relative to existing computational techniques. We also used this approach to predict all human transcription factors and found that the top-ranked proteins mostly belonged to the Krueppel C2H2-type zinc finger protein family. This family has been shown to be strongly associated with DNA methylation, and some members have been shown to bind preferentially. Taken together, these findings highlight the practical value of our two-step method for rapidly and reliably identifying transcription factors with specific methylation-binding preferences, offering a powerful complement to traditional high-throughput experiments.

## Introduction

Transcription factors (TFs) are crucial in maintaining the three-dimensional structure of the genome [1, 2]. By mediating long-range DNA interactions, TFs help to form topologically associated domains and loops [3], which subsequently influences their positioning within the nucleus and modulates transcriptional regulation [4, 5].

Traditionally, TFs are thought to bind primarily to unmethylated DNA, with methylation of CpG dinucleotides inhibiting their binding [6, 7]. However, recent experiments have identified numerous TFs that preferentially bind to methylated DNA sequences [8]. These TF-methylation-DNA interactions can initiate transcription and influence RNA splicing [9]. Systematic identification of these TFs and clarification of their functions are critical to understanding methylation-mediated biological processes [10, 11] and associated diseases [12, 13].

Experimental high-throughput methods, such as tandem mass spectrometry [14] and HT-SELEX [15], are widely used to study how TFs bind to methylated DNA [16, 17]. However, given the rapid increase in protein sequences, using these methods to annotate new TFs is resource-demanding [18, 19]. Therefore, the development of computational approaches to identify TFs that preferentially bind to certain methylated DNA (TFPMs) is crucial [20, 21]. In particular, in silico strategies hold promise for providing a cost-effective preliminary screen to help prioritize likely candidates, thereby reducing the scope and expense of subsequent wet-lab validation [22].

In 2020, Liu *et al*. developed an XGBoost approach to identify TFPMs using a dataset of TFs and TFPMs [11]. They encoded protein sequences using dipeptide composition (DC) features [23] and then applied an XGBoost algorithm [24] to determine whether the sequences represented TFPMs. They then tested the model on an independent test set with a sensitivity of 71.01% and a specificity of 64.86%. In 2022, Li *et al*. used a skip-gram model [25] to transform each sequence into a tripeptide word vector [26], which was then entered into a Long Short-Term Memory model [27] to classify TFPMs [28]. They achieved a sensitivity of 78.26% and a specificity of 64.87%. Also in 2022, Nguyen *et al*. used the employed reduced dimensional G-gap DC feature [29] and an support vector machine (SVM) algorithm with a sensitivity of 82.61% and a specificity of 64.86% [30]. However, current methods often excessively rely on relatively small-scale or highly homologous datasets, leading to insufficient evaluation of their performance when faced with novel species or previously unseen sequences. Consequently, their generalizability and applicability in broader biological contexts remain questionable.

In this study, we report a two-step method to improve the classification performance of TFPMs. First, we used a fine-tuned ProtBERT-BFD [31] model to classify the TFs. Second, we combined the reduced amino acid category [32] and K-mer method [30] to represent protein sequences. These extracted features vectors are then input into an SVM model [24] for classification. This two-step approach utilizes two different models to address specific tasks. To ensure direct comparability with previous work, we use the same datasets as in previous studies and our results indicate that our method outperforms in various metrics. Furthermore, we performed comprehensive cross-species validation with mouse dataset. Two experiments were designed to systematically compare the performance of our method with existing approaches. In addition, we validated our approach using a large-scale independent dataset comprising 1639 human transcription factors. Some indirect evidence was found to support our method applicability. Together, these extensive evaluations substantiate the robustness and reliability of our method.

## Materials and methods

### Datasets

**Fig 1:**
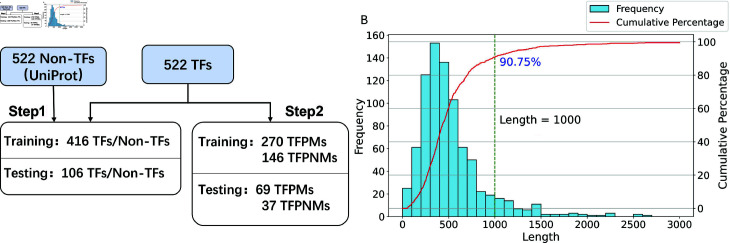
Dataset Processing Steps and Distribution. (A) Step 1 creates training and testing datasets for TFs and non-TFs, and Step 2 creates training and testing datasets for TFPMs and TFPNMs. (B) The frequency distribution and cumulative percentage of protein sequence lengths.

The datasets are sourced from Liu *et al*. [11]. These datasets include the TFs datasets and the TFPMs datasets originally curated by Wang *et al*. [18] and Yin *et al*. [33]. The method for dividing the dataset is shown in Fig 1A. Wang *et al*. manually collected TFPMs data from 601 human and 129 mouse samples. Yin *et al*. performed experimental studies to understand the influence of DNA methylation on TF-methylation-DNA binding and identified 286 TFs with a preference for non-methylated DNA (TFPNMs).

The following criteria are used for processing TFPMs and TFNMs:

1. Removal of sequences with ambiguous amino acid residues (‘B’, ‘X’, or ‘Z’).
2. Application of the CD-HIT tool [15] with a 25% similarity threshold to eliminate redundant sequences.

This preprocessing procedure yielded 522 TFs, which were divided into 270 TFPMs and 106 TFPNMs for the training dataset, with 69 TFPMs and 37 TFPNMs assigned to the independent test set. The selection of the 522 non-TFs was based on stringent screening from the UniProt 2019_11 database [34], adhering to the following rules:

1. Proteins must be peer-reviewed.
2. Proteins should be proven to exist with protein level evidence.
3. Only full-length proteins are included.
4. Proteins should contain more than 50 amino acids in length.
5. Exclusion of proteins that exhibit DNA-binding TF activity.
6. Proteins must be from humans.

The training dataset included 416 TFs and 416 non-TFs, with an independent test set containing 106 TFs and 106 non-TFs. These datasets remain among the highest-quality and most comprehensive publicly available resources specifically documenting TFs with known methylation preferences [11]. To further reduce noise and enhance generalizability, we applied strict data cleaning protocols. Removing sequences with ambiguous amino acids or high sequence similarity is to reduce noise and enhance generalizability. After an extensive literature search, we have not identified any larger-scale public collections dedicated to TF methylation preference, making these the most viable datasets for both training and benchmarking. For additional validation, our study also tested performance on an independent mouse dataset from the same source, where the proposed approach continued to outperform existing methods. Although the dataset size is relatively small, fine-tuning on these sequences (combined with large-scale pre-training in the first step) was sufficient for our core analyses (with figures of various metrics during fine-tuning available in our public repository). In the future, we plan to incorporate newly available data to further refine and expand the model’s capabilities.

### Identifying TFs

**Fig 2:**
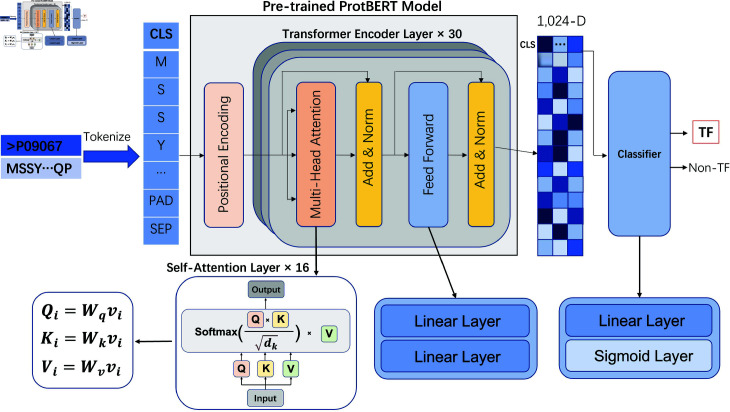
Flowchart for Identifying TFs. The sequences are tokenized and encoded, followed by processing through the ProtBERT model. The final CLS token embedding is used for the binary classification of TFs and non-TFs.

Our methodology for identifying TFs is illustrated in Fig 2. Our approach begins with the tokenization of amino acid sequences, followed by the fine-tuning of the ProtBERT model specifically for the task of TF prediction. Finally, we evaluate the performance using a comprehensive set of metrics.

#### Amino acid tokenization.

Proteins can be analyzed through the framework of natural language processing (NLP) models. The alphabet for these sequences comprises 20 natural amino acids and ‘X’ for non-standard or unseen amino acids. Each amino acid within the sequence is considered a word or token, and each protein sequence is beginning with a special class token (CLS) [35]. This CLS token summarizes the features of all tokens in the input sequence [36], allowing the use of contextual embeddings provided by the ProtBERT model.

The experimental results suggest that a maximum sequence length (max_len) of 1000 gives the optimal AUC (Table 1). Sequences shorter than this length are padded with PAD tokens, while longer sequences are truncated. In addition, a separator (SEP) token is appended to the end of each sequence to indicate its boundary. Each sequence is standardized to 1000 tokens, subsequently then transformed into 1,024-dimensional vectors by the ProtBERT model.

**Table 1:** Performance Metrics for Different Max_len Values.

Max_len	Sensitivity	Specificity	Accuracy	MCC	AUC
500	0.9151	0.9340	0.9245	0.8492	0.9486
750	0.9245	0.9528	0.9387	0.8777	0.9726
**1000**	**0.9340**	**0.9433**	**0.9387**	**0.8773**	**0.9801**
1250	0.9340	0.9151	0.9245	0.8492	0.9703
1500	0.9434	0.9151	0.9292	0.8588	0.9553

The selection of 1000 as the maximum protein sequence length is justified by the cumulative distribution of sequence lengths in our dataset (Fig 1B). At this length, the cumulative percentage reaches 90.75%, covering a significant portion of the dataset, which ensures that the model can learn appropriate patterns. Excessive padding can cause noise, while insuï¬€icient sequence information can impair the model’s learning ability.

#### Fine-tuning for the TFs prediction.

ProtBERT is a protein-specific variant of the BERT (Bidirectional Encoder Representations from Transformers) model that has been pre-trained on a massive corpus of approximately 2.1 billion sequences sourced from the Big Fantastic Database, including UniProt/TrEMBL + SwissProt [34], Metaclust and others. The primary advantage of ProtBERT, deep understanding of protein language, is acquired from the vast amount of data it was trained on [31]. We use the Hugging Face Transformers API to simiplify the fine-tuning of model.

In this context, each amino acid and each peptide is treated like a word or a sentence. Sinusoidal positional encoding is employed to give each token a positional context, while the pre-trained model uses a mechanism of self-attention to capture hierarchical information and relationships between words in the input data. The final layer is then used for supervised learning specifically for fine-tuning.

The ProtBERT model architecture is composed of several transformer encoders layers. Each transformer encoder layer is equipped with multi-head attention mechanisms, feed-forward networks, and normalization steps for the layers. The self-attention mechanism in each encoding layer computes the attention scores using the Query, Key and Value matrices, as shown in Fig 2. These attention scores are then used to weigh the importance of each token in the sequence and capture complex patterns that are important for accurate classification of the sequence [37].

During the fine-tuning phase, we extract the CLS token embedding for the downstream task. This is different from the standard fine-tuning method of ProtBERT for sequential tasks, where the embeddings generated by the final hidden state of BERT are pooled in the global average [38]. Comparative tests against alternative pooling strategies (mean pooling and max pooling) revealed that CLS-based embeddings achieved higher Accuracy, Matthews Correlation Coefficient (MCC), Specificity, and F1-score (Table 2). This superior performance likely arises from the CLS token’s dedicated role in Transformer models: it collects contextual information from every token in the sequence, yielding an enriched representation reflective of the protein’s overall semantics. Because protein sequences share fundamental parallels with natural language data, harnessing the CLS token is particularly advantageous for capturing globally relevant features (such as conserved domains or characteristic amino acid distributions) , which is necessary for TF identification. In contrast, uniform (mean) or selective (max) pooling can dilute these key contextual patterns [39].

**Table 2:** Comparison results of different embeddings.

	ACC	Sensitivity	Specificity	MCC	AUC	F1
**CLS**	**92.92**	**94.34**	**91.57**	**85.88**	96.02	**93.02**
**Mean**	92.45	94.34	90.57	84.97	95.85	92.59
**Max**	92.45	94.34	90.57	84.97	**97.49**	92.59

For binary classification of TFs and non-TFs, where each input is labeled 1 and 0, respectively, we use a linear layer and a sigmoid layer to compute the probability that the CLS token vector is a TF. Predictions with probabilities of 0.5 or higher were classified as TFs, while those with probabilities less than 0.5 were non-TFs. The binary cross-entropy loss function is commonly used for binary classification problems [40], defined as follows :

$$L(y,\hat{y})=-\frac{1}{N}\sum_{i=1}^{N}\left[y_{i}\log({\hat{y}}_{i})+(1-y_{i})\log(1-{\hat{y}}_{i})\right]$$ (1)

where *y*_*i*_ represents the true label, which can be either 0 or 1, and ${\hat{y}}_{i}$ is the predicted probability for the *i*-th input data belongs to the positive class. We evaluate the performance using an independent test set. The results prove that our method outperforms the existing methods across all criteria.

### Identifying TFPMs

**Fig 3:**
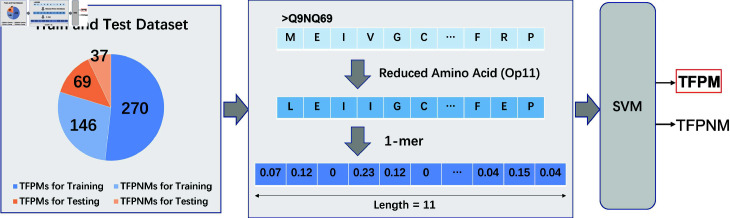
Flowchart for Identifying TFPMs. Protein sequences are first encoded using RAAC method, which groups amino acids into clusters to reduce complexity. These reduced sequences are then processed using the K-mer counting approach, resulting in frequency vectors. These vectors are fed into an SVM model, effectively distinguishing TFPMs from TFPNMs.

In this section, we present our method to identify TFPMs as shown in Fig 3. First, we apply RAAC to group amino acids and summarize the protein sequences. Next, we use K-mer counting to divide these sequences into overlapping subsequences, which captures important local patterns. Finally, we input these encoded sequences into a carefully tuned SVM model to optimize classification performance. Unlike the initial ProtBERT-based approach for TF classification, we chose not to employ an end-to-end ProtBERT model for TFPM detection. The rationale is twofold: practical constraints and the inherent limitations of large Transformer architectures, which can be prone to overfitting and inconsistent performance when dealing with smaller datasets or distinguishing subtle biological variations (e.g., TFPMs vs. TFPNMs). Instead, to focus on critical local sequence motifs associated with methylation preference and mitigate overfitting, we adopted a more interpretable traditional machine learning framework. First, we applied Reduced Amino Acid Alphabet Categories (RAAC) to cluster the 20 standard amino acids according to their physicochemical or structural similarities, thereby reducing dimensionality while retaining biologically meaningful information. We then utilized K-mer counting to capture relevant local sequence patterns. Finally, the extracted feature vectors were input into a tuned Support Vector Machine (SVM) classifier, achieving reliable and consistently strong classification outcomes.

#### Reduced amino acid category.

In RAAC, the standard amino acids are categorized into different sets, each represented by a unique symbol [32]. Research has proven that reducing the complexity of sequences minimizes information redundancy and improves the computational performance of machine learning methods [41]. The key to RAAC’s effectiveness lies in how the amino acids are grouped.

In their study, Etchebest *et al*. [42] identified 16 protein blocks (PBs) from the three-dimensional structures of proteins composed of different amino acid blocks. They encoded the PDB-REPRDB dataset [43] using these 16 PBs into PB sequences and created a 20 × 15 matrix of amino acid occurrences for each PB. These matrices were then transformed and combined into a final 20 × 240 matrix. To analyze the relationships between amino acids, they measured the distances among them and employed R software for clustering and further analysis. This process resulted in a clustering scheme (Op 11) where the amino acids were assigned to 11 clusters (Table 3). We use the Op11 scheme to simplify the sequences.

**Table 3:** Op(11) Grouping for Amino Acids.

Index	Group of Amino Acids	Symbols
1	G (Glycine)	G
2	I (Isoleucine), V (Valine)	I
3	F (Phenylalanine), Y (Tyrosine), W (Tryptophan)	F
4	A (Alanine)	A
5	L (Leucine), M (Methionine)	L
6	E (Glutamic acid), Q (Glutamine), R (Arginine), K (Lysine)	E
7	P (Proline)	P
8	N (Asparagine), D (Aspartic acid)	N
9	H (Histidine), S (Serine)	H
10	T (Threonine)	T
11	C (Cysteine)	V

#### K-mer counting-based features.

Following the RAAC method, we used the K-mer approach to encode the protein sequences. Previous research has shown that diverse K-mer-based sequence encoding methodologies can yield robust performance in related tasks. For example, Liu *et al*. [11] found that dipeptide composition (2-mers) outperformed other encodings such as SAAC and CTD, whereas Li *et al*. [28] used tripeptide word vectors (3-mers), and Nguyen *et al*. [30] adopted a G-gap dipeptide approach closely aligned with K-mer representations. Drawing on these insights, we systematically tested different K values to identify the optimal configuration for our particular dataset and classification goals. In the K-mer method, protein sequences are decomposed into overlapping subsequences of length K, where each K-mer represents a contiguous sequence of K amino acids [25]. By analyzing the frequency of each K-mer within a protein sequence, we captured local sequence patterns and features that are important for distinguishing TFPMs and TFPNMs.

To calculate the frequency of each K-mer, we move a sliding window of length K across the protein sequence. At each position, we count the occurrence of each K-mer. For example, if K=3, the sequence ‘ACDEFG’ would be divided into ‘ACD’, ‘CDE’, ‘DEF’ and ‘EFG’. The result of this process is a frequency vector that represents the number of all possible K-mers.

This combined RAAC and K-mer encoding method effectively strategically mitigates the complexity of the sequence while preserving crucial information for classification [44]. The frequency vectors generated from the K-mer are then used as input features for our machine learning model, providing a comprehensive feature set that balances sequence complexity and information richness [45].

#### SVM model.

The final step in our method, which combines RAAC and K-mer encoding, is the use of an SVM model. The SVM model works by determining the optimal hyperplane that most effectively distinguishes between different classes in a high-dimensional space.

The computational pipeline for TFPM classification begins with the encoding of protein sequences using the RAAC and K-mer methods. The RAAC method reduces the complexity of protein sequences by grouping amino acids, while the K-mer approach splits the sequences into overlapping subsequences of length K and thus captures local sequence patterns [46]. These methods generate frequency vectors that serve as input features for the SVM model to identify TFPMs.

Finally, we validate the model with optimized parameters on an independent test set. The results demonstrates that our model outperforms existing models on several metrics and confirm the effectiveness of our RAAC + K-mer + SVM approach in identifying TFPMs.

We use 5-fold cross-validation and the independent datasets to evaluate the performance of our model comprehensively. These methods are standard for verifying the accuracy and reliability of predictive models. We use four primary statistical metrics to evaluate the performance: Sensitivity, Specificity, Accuracy, and Matthews Correlation Coefficient (MCC):

$$\text{Sn}=\frac{\text{TP}}{\text{TP}+\text{FN}}$$ (2)

$$\text{Sp}=\frac{\text{TN}}{\text{TN}+\text{FP}}$$ (3)

$$\text{Acc}=\frac{\text{TP}+\text{TN}}{\text{TP}+\text{FP}+\text{TN}+\text{FN}}$$ (4)

$$\text{MCC}=\frac{\text{TP}\times \text{TN}-\text{FP}\times \text{FN}}{\sqrt{\left(\text{TP}+\text{FN})(\text{TN}+\text{FP})(\text{TP}+\text{FP})(\text{TN}+\text{FN}\right)}}$$ (5)

where FP denotes false positives, FN denotes false negatives, TP denotes true positives and TN denotes true negatives.

Additionally, we assess the model using the Area Under ROC Curve (AUC). This metric is used to evaluate the performance of a binary classification model. The ROC curve is a graphical representation that illustrates the diagnostic ability of a classifier by plotting the Sensitivity against the 1 - Specificity at various thresholds [47]. The AUC is particularly useful with imbalanced datasets, where the number of positive and negative instances is significantly different [48]. By integrating these evaluation metrics, we ensure a robust and comprehensive analysis of the classifier’s performance and provide detailed comparisons of the model’s capabilities across different datasets and validation methods.

## Results and discussion

### TFs classification

To identify TFs, we employ a fine-tuned ProtBERT model that trained and evaluated on the same datasets as the existing studies to ensure a fair comparison. Our finetuned ProtBERT model outperforms existing methods on all key metrics, with an accuracy of 93.87%, sensitivity of 93.40%, specificity of 94.33%, MCC of 0.8773, and an AUC of 0.9801. These results showcase significant improvements over previous models such as CTD_SAAC_DC + SVM [11], Tripeptide + LSTM [28] and PSSM + CNN [30]. Table 4 summarizes evaluation results.

**Table 4:** Comparison of Methods for TF and TFPM Classification.

Problem	Method	Sensitivity	Specificity	Accuracy	MCC	AUC
TF vs Non-TF	CTD_SAAC_DC + SVM	0.8019	0.8585	0.8302	0.6614	0.9116
TF vs Non-TF	Tripeptide + LSTM	0.8868	0.8396	0.8663	0.7272	0.9130
TF vs Non-TF	PSSM + CNN	0.9056	0.8396	0.8726	0.7469	0.9596
**TF vs Non-TF**	**Ours**	**0.9340**	**0.9433**	**0.9387**	**0.8773**	**0.9801**
TFPM vs TFPNM	DC + XGBoost	0.7101	0.6486	0.6887	0.3471	0.7356
TFPM vs TFPNM	Tripeptide + LSTM	0.7826	0.6487	0.7359	0.4831	0.8324
TFPM vs TFPNM	RGDC + SVM	0.8261	0.6486	0.7642	0.4778	**0.8486**
**TFPM vs TFPNM**	**Ours**	**0.8696**	**0.6757**	**0.8019**	**0.5568**	0.8351

The large training dataset and the significant differences between TFs and non-TFs allow the model to fully utilize its powerful feature extraction capabilities. The extensive pre-training of the ProtBERT model on a large protein sequence database equips it with a robust understanding of protein structures and functions [36]. This enables the model to learn intricate patterns and relationships within protein sequences, which are further refined during the fine-tuning process [49]. The ability of the ProtBERT model to capture both global and local sequence features contributed to its exceptional performance across all evaluation metrics [31].

To optimize model performance, we test different combinations of hyperparameters for fine-tuning the model, including learning rate, weight decay, and others. The final hyperparameters that perform best in terms of AUC are 5 training epochs, a weight decay of 0.1, a learning rate of 5E-5, and no warmup steps. While other parameters such as learning rate and weight decay cause only minor fluctuations in performance with a suï¬€icient number of training epochs, max_len proves to be a critical factor that directly correlates with the effectiveness of the model. Our experimental results indicate an optimal upper limit; beyond this, additional sequence length introduces noise and may degrade performance by including irrelevant or less informative protein regions [39]. Conversely, excessively short max_len values may omit crucial contextual data required to accurately discriminate between TFs and non-TFs [50].

### TFPMs classification

We use a combination of RAAC and K-mer encoding methods with an SVM classifier. A comprehensive grid search is performed to optimize the parameters and maximize the AUC. Our RAAC + K-mer + SVM method achieved an accuracy of 80.19%, a sensitivity of 86.95%, a specificity of 67.57%, an MCC of 0.5568 and an AUC of 0.8351 (Table 4). These results confirm that our approach significantly outperforms existing models.

The second experiment faced the challenge of classifying TFPMs with a smaller training dataset and more subtle differences between TFPMs and TFPNMs. The key to distinguishing TFPMs lies in the local structure of the protein sequences, which makes the task even more complex. Large models such as ProtBERT tend to overfit in such scenarios due to the limited amount of data, as the model tries to learn the noise present in the small dataset rather than general patterns [51]. CNN and MLP neural networks are also not well suited for small datasets as they require high dimensional feature extraction results [52]. These models usually rely on a large amount of data to learn useful representations, and in the absence of suï¬€icient data, they struggle to generalize well.

To solve this problem, we introduce the RAAC method to simplify the information. The K-mer approach, inspired by DC’s effective feature extraction strategy [53], extends this concept by capturing local sequence patterns through overlapping subsequences. The effectiveness of overlapping subsequences lies in their ability to preserve contextual information, which is crucial for identifying subtle differences in local sequence structures [54]. By decomposing sequences into K-mers, we capture these local patterns without losing important contextual information.

**Fig 4:**
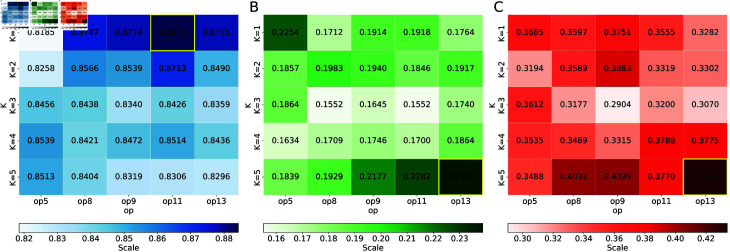
Comparison of Different Models. (A) Grid search results for the SVM model. (B) Grid search results for the RF model. (C) Grid search results for the XGboost model.

To optimize the SVM classifier, we performed a comprehensive grid search over several parameters (Fig 4A), including the number of RAAC groups (op), the K-mer length (K), and the SVM hyperparameters (C and gamma). Specifically, we evaluate op values of 5, 8, 9, 11, and 13; K values of 1, 2, 3, 4, and 5; along with different settings for C and gamma. The optimal parameters are Op = 11, K = 1, SVM-C = 0.01 and SVM-gamma = 1, which provide the best performance in terms of the AUC. The evaluation results on independent datasets show that our method outperforms the existing models.

Although setting K=1 might seem to risk losing valuable dipeptide or tripeptide information, RAAC’s grouping of the 20 amino acids by physicochemical properties helps retain crucial functional and structural signals. In effect, K=1 encoding in RAAC captures the frequency of these simplified amino acid categories, preserving key motif signatures while drastically reducing dimensionality. This implies a biologically meaningful connection between methylation preferences and single-residue-level physicochemical traits, such as charge or hydrophobicity. We evaluated K values from 1 to 5, observing that higher K values substantially increased feature dimensionality without improving predictive performance, ultimately risking overfitting and obscuring important biological cues. Overall, these experiments support our conclusion that K=1 optimally balances dimensionality reduction with the preservation of meaningful sequence patterns, underscoring the link between methylation specificity and single-residue features.

By comparing the performance of SVM with that of other classical models, such as XGBoost [53] and Random Forest (RF) [30], we found that only SVM performed reliably (Fig 4). This can be attributed to the inherent characteristics of SVM that are effective in high-dimensional spaces [55]. It is able to find an optimal hyperplane that maximizes the margin between the classes. By using the RBF kernel, the ability to capture non-linear relationships within the data is further enhanced [24]. These properties make the SVM particularly robust for our task.

### Generalization of TFPM model to new data

To assess the robustness and generalizability of our model, we test its predictive performance using independent datasets not used before. We conducted a cross-species validation with 129 mouse TFPM sequences in two complementary experiments: (1) treating the mouse TFPMs as known transcription factors to isolate TFPM identification, and (2) treating them as general protein sequences to assess the full two-step pipeline. Although definitive negative samples of mouse TFs without methylation preferences were unavailable, these experiments closely approximate real-world conditions. For existing methods, we used publicly available servers (where active) or replicated their inference procedures from the original source code. As shown in Table 5, our model consistently surpassed all baselines for both TF and TFPM classification, reinforcing its cross-species applicability and providing a comprehensive quantitative benchmark. Detailed information on the identified sequences can be found in our publicly available GitHub repository. In addition, we compiled a comprehensive dataset of 1,639 human transcription factors from public databases [56], where we exclude 522 TFs known for specific binding preferences to minimize bias. Using optimal hyperparameters (Op = 11, K = 1, SVM-C = 0.01, SVM-gamma = 1) determined by 5-fold cross-validation, we trained model to predict TFPM on this new dataset. We focused on the top 20 proteins with the highest TFPM probabilities, which are listed in Table 6.

**Table 5:** Cross-species generalization results.

	Liu	Nguyen	Li	Ours
**Exp1-TFPM**	66	80	84	**122**
**Exp2-TF**	55	48	45	**72**
**Exp2-TFPM**	21	27	28	**67**

**Table 6:** Prediction on unknown sequences.

Index	UniProtID	Gene	Protein Name	Organism	Protein Family	Probability
1	P52926	HMGA2	HMGI-C	Homo sapiens	HMGA	0.7064
2	P17096	HMGA1	HMG-I/HMG-Y	Homo sapiens	HMGA	0.6999
3	Q86XF7	ZNF575	Zinc finger protein 575	Homo sapiens	Krueppel C2H2-type Zinc-finger	0.6940
4	Q8N972	ZNF709	Zinc finger protein 709	Homo sapiens	Krueppel C2H2-type Zinc-finger	0.6906
5	Q8N8L2	ZNF491	Zinc finger protein 491	Homo sapiens	Krueppel C2H2-type Zinc-finger	0.6867
6	P17017	ZNF14	Zinc finger protein 14	Homo sapiens	Krueppel C2H2-type Zinc-finger	0.6851
7	Q9UK33	ZNF580	Zinc finger protein 580	Homo sapiens	C2H2-type Zinc-finger	0.6850
8	Q9P2F9	ZNF319	Zinc finger protein 319	Homo sapiens	Krueppel C2H2-type Zinc-finger	0.6828
9	Q96CX3	ZNF501	Zinc finger protein 501	Homo sapiens	Krueppel C2H2-type Zinc-finger	0.6816
10	Q15651	HMGN3	HMGN3	Homo sapiens	HMGN	0.6805
11	P0CG24	ZNF883	Zinc finger protein 883	Homo sapiens	Krueppel C2H2-type Zinc-finger	0.6802
12	P0CG23	ZNF853	Zinc finger protein 853	Homo sapiens	Krueppel C2H2-type Zinc-finger	0.6792
13	Q499Z4	ZNF672	Zinc finger protein 672	Homo sapiens	Krueppel C2H2-type Zinc-finger	0.6783
14	Q8TF45	ZNF418	Zinc finger protein 418	Homo sapiens	Krueppel C2H2-type Zinc-finger	0.6780
15	Q9P0T4	ZNF581	Zinc finger protein 581	Homo sapiens	Krueppel C2H2-type Zinc-finger	0.6775
16	Q6ZR52	ZNF493	Zinc finger protein 493	Homo sapiens	Krueppel C2H2-type Zinc-finger	0.6769
17	Q7Z7K2	ZNF467	Zinc finger protein 467	Homo sapiens	Krueppel C2H2-type Zinc-finger	0.6758
18	Q7Z340	ZNF551	Zinc finger protein 551	Homo sapiens	Krueppel C2H2-type Zinc-finger	0.6755
19	Q05481	ZNF91	Zinc finger protein 91	Homo sapiens	Krueppel C2H2-type Zinc-finger	0.6752
20	P17026	ZNF22	Zinc finger protein 22	Homo sapiens	Krueppel C2H2-type Zinc-finger	0.6751

Most of these top predictions are from the Krueppel C2H2-type Zinc-finger family. These proteins are particularly effective at interacting with methylated CpG islands [57], which are important for regulating gene expression[58]. Further literature review revealed that proteins of the Krueppel C2H2-type Zinc-finger family, such as Kaiso, have been shown to exhibit binding preferences [59]. Kaiso binds to methylated DNA preferentially, recognizes methylated CpG islands and attaches to them via its zinc finger motifs to repress gene transcription [60].

These results underpin the robustness of our model and indicate that it can reliably predict TFPMs. The predictive power of the model holds great potential for future research into the binding preferences of this protein family for methylated DNA and could provide new insights into gene regulation and epigenetic modifications. All test results of the models on multiple parameters, as well as the prediction results of this method on unseen data, are included in S1 Zip. The filtered dataset obtained from all human transcription factors, along with previously related datasets, is provided in S2 Zip. Additionally, all codes related to this paper are publicly available at https://github.com/LiZaiyuan0619/RKmer-SVM4TFPM/.

## Conclusion

TFs are crucial for activation of genes and the organization of genome structure. Recent studies have confirmed their capacity to bind to methylated DNA, yet mechanisms behind this interaction remain unclear. Traditional experimental methods to identify TFs and TFPMs are resource-demanding. With the increasing number of protein sequences, there is a growing need for eï¬€icient computational methods to bridge the gap between annotated and non-annotated proteins.

We present a novel machine learning method to predict TFs and TFPMs. Initially, we use a fine-tuned ProtBERT model to encode these sequences and employ a classifier for the downstream task. For the classification of TFPMs, we combine RAAC and K-mer methods to represent protein sequences, and then classify these feature vectors using an SVM. Our methods are evaluated using the same datasets as in previous studies. Comprehensive performance assessment show superior accuracy, sensitivity, and specificity compared to existing models. By narrowing large candidate pools to more manageable sets, this pipeline enables researchers to allocate resources more strategically. While our primary focus is on human TFs, the methodology is readily adaptable to broader proteomes and additional species.

Future research will focus on exploring more advanced deep learning architectures and the relationship between Krueppel C2H2-type Zinc-finger family and methylated DNA. This exploration aims to further enhance the performance of our methods and gain a deeper understanding of TF and TFPM identification and the interpretation of the Krueppel C2H2-type Zinc-finger family’s binding preference for methylated DNA.

## Supporting information

S1 Zip Experiment logs and prediction results. This archive contains four files: grid search logs for the SVM, XGBoost, and Random Forest models used in TFPM classification, as well as the final prediction results of TFPM probabilities across the complete set of human transcription factors.
(ZIP)

S2 Zip Datasets. This archive provides all datasets involved in our experiments, including training and test sets for TF and TFPM classification tasks, as well as the full human transcription factor dataset used for generalization analysis.
(ZIP)
